# Spontaneous coronary artery dissection in a young patient with antiphospholipid syndrome

**DOI:** 10.1186/s12959-023-00573-5

**Published:** 2024-01-17

**Authors:** Ai Phi Thuy Ho, Eirik Tjønnfjord, Oliver Meyerdierks, Ellen Elisabeth Brodin

**Affiliations:** 1 Department of Cardiology, Kalnes Hospital, Kalnesveien 300 Grålum, P.O. Box 300, 1714 , Sarpsborg, Norway; 2 Department of Emergency Medicine, Kalnes Hospital, Sarpsborg, Norway; 3 Department of Cardiology, Akershus University Hospital, Lørenskog, Norway; 4 Department of Hematology, Akershus University Hospital, Lørenskog, Norway

**Keywords:** Antiphospholipid syndrome, Percutaneous coronary intervention, Spontaneous coronary artery dissection, Chest pain

## Abstract

A 28-year-old man diagnosed with triple positive antiphospholipid syndrome (APS) and undergoing warfarin experienced three separate admissions to the cardiac ward within a one-month period due to escalating chest pain. While the initial two admissions revealed normal results in cardiological investigations, such as blood tests, electrocardiogram, and echocardiography, the third admission unveiled signs of ST-elevation myocardial infarction (STEMI), despite the patient maintaining an INR (International Normalized Ratio) of 4. Subsequent percutaneous coronary intervention (PCI) exposed spontaneous coronary artery dissection (SCAD) of type 3. Faced with hemodynamic instability and worsening symptoms, the patient underwent stenting and was prescribed dual antiplatelet therapy in addition to warfarin. A follow-up evaluation one month later indicated a normalization of his condition.

## Introduction

Antiphospholipid syndrome (APS) is a rare autoimmune disease with an incidence of approximately 5 cases per 100,000 per year. It can manifest either as a primary disease or secondary to connective tissue diseases, particularly systemic lupus (SLE). APS is recognized as the most thrombogenic acquired form of thrombophilia and is linked to both venous and arterial thrombosis, including microthrombosis, along with complications during pregnancy. Recent years have witnessed a growing emphasis on non-criteria manifestations [1]. This case report centers on a potential complication of APS, spontaneous coronary artery dissection (SCAD). Although case reports have suggested a link between SCAD and autoimmune diseases, especially SLE, a definitive association remains elusive. The objective of this case study is to elucidate a plausible link between two rare diseases, APS and SCAD, and to highlight the complexities in treatment when they coexist.

A 28-year-old man was diagnosed with APS in 2020 due to recurrent pulmonary embolism. He exhibited strong positivity for lupus anticoagulant, anti-beta2-glycoprotein IgG, and anti-cardiolipin antibody IgG, thereby classifying him as triple positive for APS [2]. No underlying systemic disease was identified. He initiated treatment with warfarin and was instructed to maintain an INR target range of 2–3. However, due to international ratio (INR) discrepancy between capillary and venous samples, measurements were exclusively conducted at the hospital laboratory. Due to frequent anxiety, the patient consistently maintained INR levels higher between 3–4.

In October 2022, the patient was admitted to the cardiac department after his general practitioner (GP) randomly measured his troponin-T (TnT) level, which was found to be 92 ng/L (reference range < 14 ng/L). The patient had no cardiac symptoms and his venous sample showed an INR of 2.1. Electrocardiogram (ECG) revealed sinus rhythm with isolated T inversion in lead III but was otherwise normal. Comprehensive examinations, including transthoracic echocardiography (TTE), stress ECG, and magnetic resonance imaging (MRI) of the heart, all yielded normal results. Given the absence of cardiac symptoms, spontaneously normalizing TnT values, and normal imaging, he was subsequently discharged without any follow-up.

**Fig. 1:**
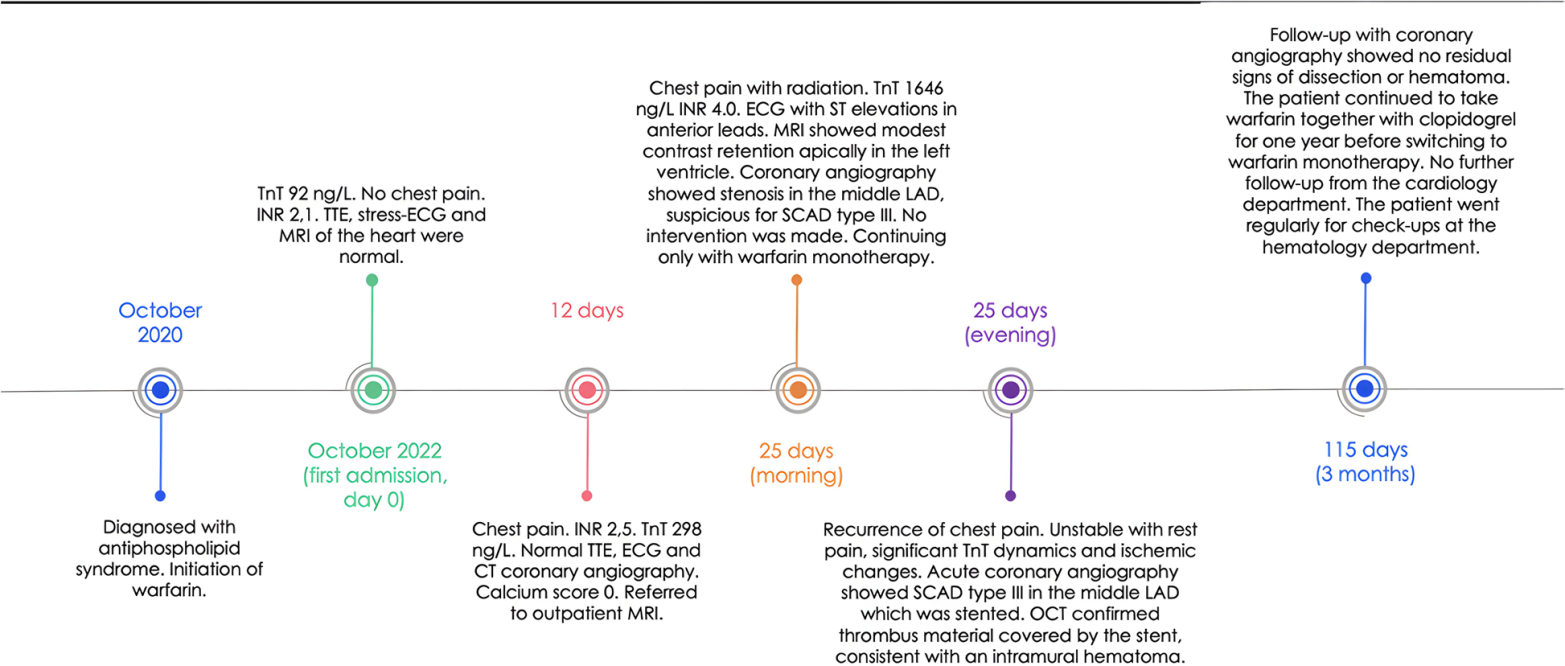
A overview of the patient´s timeline from APS (antiphospholipid syndrome) diagnosis through the acute coronary syndrome event and diagnostic and therapeutic interventions. TnT: troponin T, reference range < 14 g/L. INR: International normalized ratio

The patient was readmitted to the hospital 12 days later, reporting chest pain radiating to the left arm. Although the electrocardiogram (ECG) showed no new changes, there was a significant increase in the TnT level, peaking at 298 ng/L. D-dimer was negative. The patient had recently switched to therapeutic doses of low molecular weight heparin (LMWH) in anticipation of an overseas trip. The shift to LMWH aimed to avoid INR fluctuations in warmer regions and due to the limited possibility of measurement. LMWH was initiated when the INR was 2.1. The patient had already experienced chest pain before the transition. However, due to worsening of his chest pain, the trip was canceled, and the patient reverted to warfarin. The conversion to warfarin was conducted with close follow-up, involving 5 days of overlapping treatment and cessation when the INR reached 2.3. The patient did not miss any injection. Transthoracic echocardiography (TTE) showed no changes. Throughout the observation period, the patient remained pain-free, and telemetry revealed no arrhythmias. He underwent a CT coronary angiography, revealing normal coronary arteries with a calcium score of 0. The case was deliberated in the collegium, and he was discharged for an outpatient MRI of the heart to rule out possible myocardial damage or previous myocarditis (Fig. 1).

**Fig. 2:**
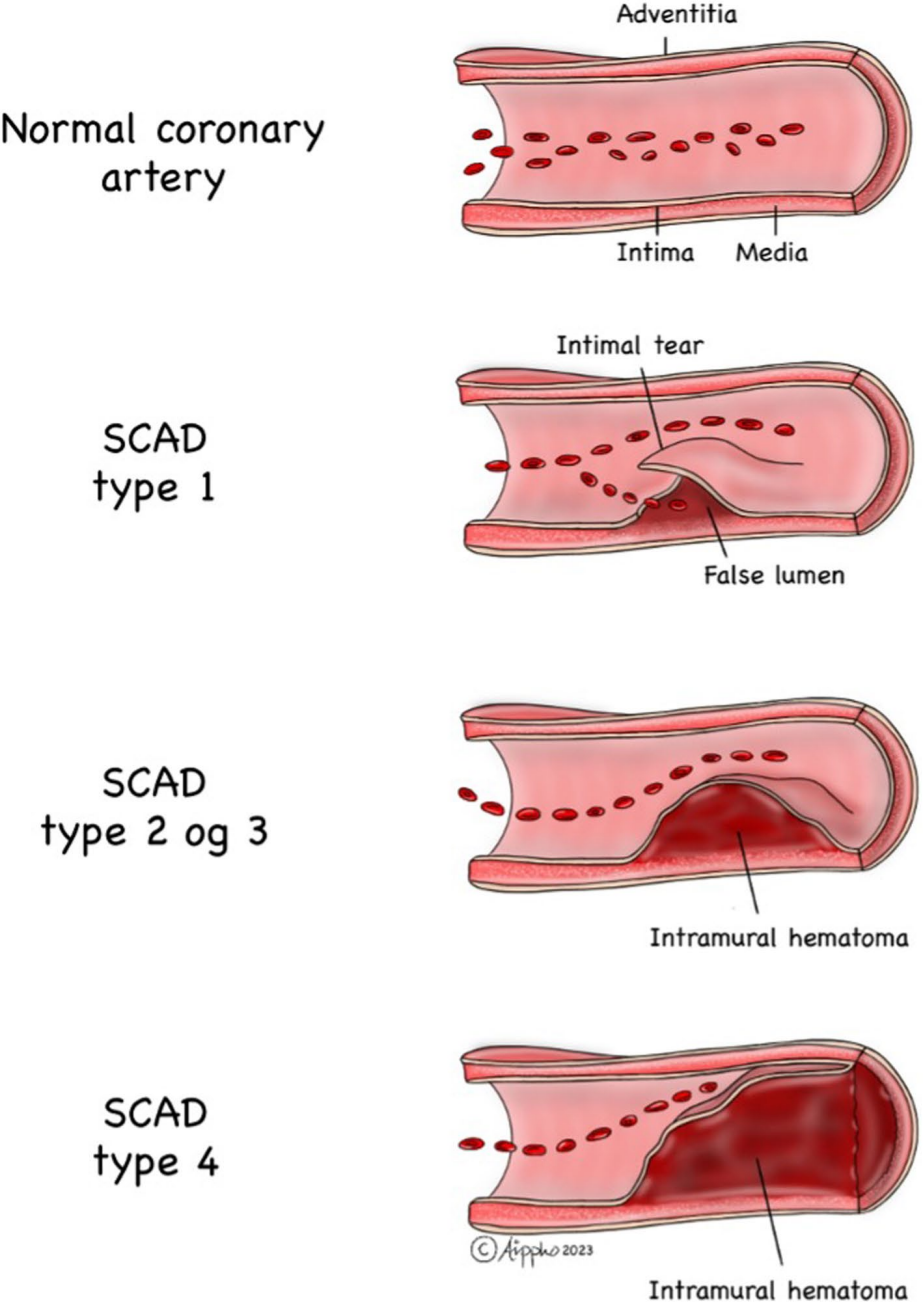
It requires OCT (optical coherence tomography) or IVUS (intravascular ultrasound examination) to be able to make the correct diagnosis. SCAD (spontaneous coronary artery dissection) type 1 is due to an intimal tear that separates the true lumen from the false lumen. SCAD types 2 and 3 are characterized by the absence of an intimal tear and appear as a long segment of diffusely narrowed artery due to an intramural hematoma causing stenosis of varying severity. SCAD type 4 results from complete occlusion of the vessel

**Fig. 3:**
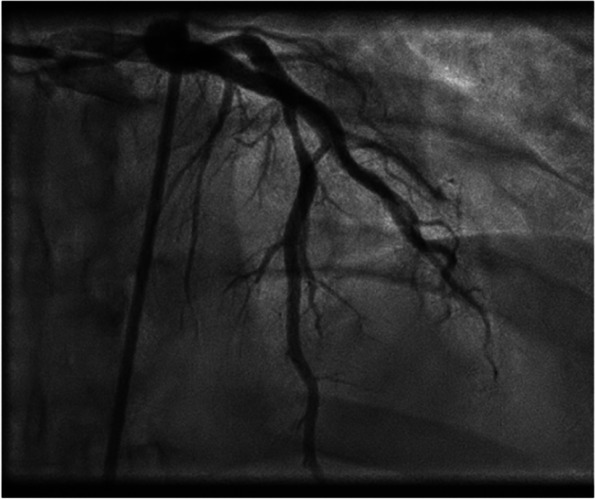
Coronary angiography with confirmed SCAD (spontaneous coronary artery dissection) type 3 in the LAD (left anterior descending artery)

Thirteen days later, the patient was readmitted to the hospital due to chest pain radiating to both arms. TnT values significantly increased to 1646 ng/L, and the INR was 4.0. The ECG showed new ST elevations in the anterior leads, consistent with STEMI. The outpatient MRI conducted just before admission revealed possible modest contrast retention apically in the left ventricle. Despite the elevated INR, urgent coronary angiography was performed on vital indication, revealing stenosis in the middle left anterior descending artery (LAD), while all other arteries were open. The findings were suggestive of spontaneous coronary artery dissection (SCAD) type 3 (as shown in Fig. 2). After discussion between cardiologists and hematologists, it was decided not to intervene immediately since the patient was stable. Instead, the approach involved waiting with double platelet inhibition (DAPT), but continue with monotherapy treatment using warfarin. The same evening, the patient again experienced chest pain, alleviated by intravenous nitroglycerin infusion. The ECG showed slightly increasing ST elevations in the anterior wall, and a new acute coronary angiography confirmed SCAD type 3 in the middle LAD (as shown in Figs. 2 and 3) based on typical image of focal/tubular stenosis. The patient was consistently covered with full dose anticoagulation and maintained an appropriate INR level, minimizing the likelihood of a thrombotic event.

**Fig. 4:**
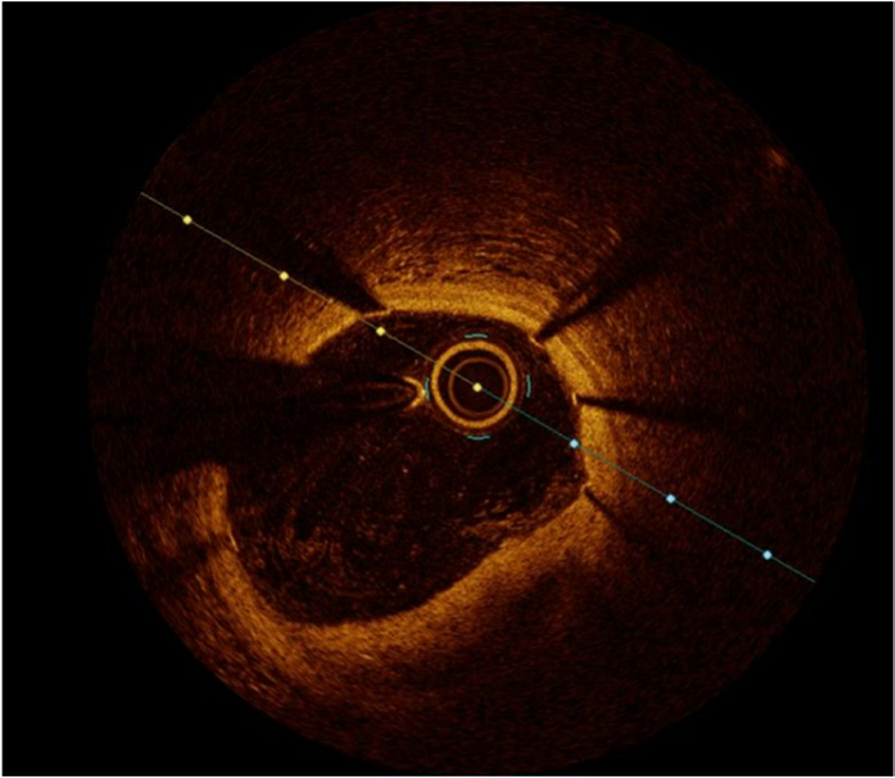
OCT (optical coherence tomography) showing intramural hematoma

**Fig. 5:**
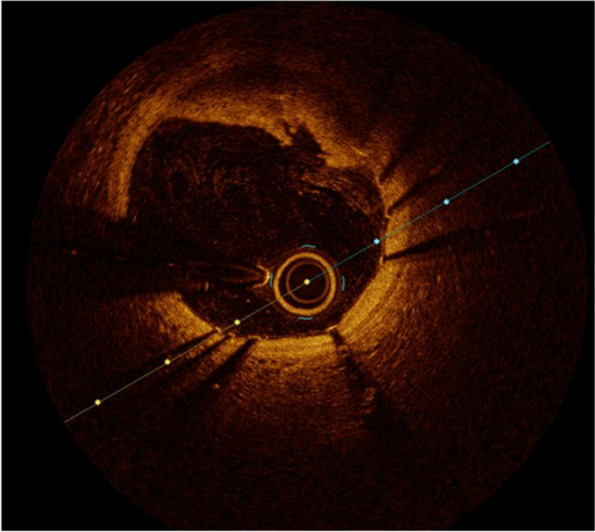
OCT (optical coherence tomography) showing dissection with thrombus masses

The patient's condition was unstable, characterized by rest chestpain, significant TnT dynamics, and ischemic changes, prompting the placement of a stent in the middle LAD. Subsequent optical coherence tomography (OCT) revealed thrombus material covered by the stent, consistent with an intramural hematoma. Additionally, an intimal tear was detected, which was also covered by the stent, as shown in Figs. 4 and 5.

After a multidisciplinary meeting involving cardiologists and hematologists, the decision was to add DAPT to warfarin, along with angiotensin-converting (ACE) inhibitors, statins, and beta blocker. The INR target was set to 2.5. TTE revealed hypokinesia in the distal part of the septum, extending to the apex and inferior wall. The ejection fraction (EF) was 48%. The patient remained hemodynamically stable throughout the observation period and was discharged with plans for follow-up at the hematology outpatient clinic, as well as a scheduled outpatient coronary angiography. Angiography performed three months later showed positive outcomes following percutaneous coronary intervention (PCI) of the LAD, with no residual signs of dissection or hematoma. The patient continued on warfarin along with clopidogrel for one year before transitioning to warfarin monotherapy. No OCT control was performed. There was no additional follow-up from the cardiology department after this point, but the patient regularly attended check-ups at the hematology department. Here the patient was retested for the possibility of vasculitis, with negative results for markers of ANA, complement and inflammatory markers, indicating a low likelihood of vasculitis.

The APS tests were repeated to determine if an increase in antibody titers could signify heightened disease activity and contribute to increased autoimmunity. The samples consistently displayed elevated antibodies. Consequently, the patient was not administered immunosuppressive treatment.

## Discussion

In recent years, SCAD has exhibited an increasing incidence as a cause of heart attacks, particularly among women aged 47–53 and in pregnant women [3]. SCAD is a non-atherosclerotic cause of coronary artery dissection, presenting either as classical dissection with a visible false lumen or as an intramural hematoma, potentially accompanied by an intimal tear. OCT has played a crucial role in supporting two hypotheses: that the primary event could be an intimal dissection with media involvement or a medial intramural hematoma in the tunica media due to vaso vasorum bleeding [4]. The development of myocardial infarction results from coronary obstruction caused by luminal compression, either from dissection or the spread of intramural hematoma (Fig. 2).

While SCAD can occur in all coronary arteries, the LAD and its diagonal branches are predominantly affected. Although the majority of SCAD lesions can be diagnosed by angiography alone, distinguishing potential SCAD from other causes of coronary artery stenosis can be challenging. Especially focal intramural hematoma of type III may be misinterpreted as atherosclerotic lesions. When angiography fails to diagnose SCAD, the use of intravascular imaging such as intravascular ultrasound (IVUS) or OCT should be considered. OCT can reveal true and false lumen, intramural hematoma, dissection and intimal tear. Intravascular imaging may also aid in ruling out other causes of coronary artery stenosis, including atherosclerotic plaque. However, it is crucial to note that there is a risk of increasing the dissection during intracoronary instrumentation. If the patient is stable and symptom-free, a conservative treatment approach is therefore preferable to an invasive one (5, algorithm).

The underlying mechanism of SCAD is not fully understood. Some studies suggest a connection between autoimmune diseases, particularly SLE and SCAD [5, 6]. However, a definite connection has not been demonstrated. Registry data indicate a low incidence of systemic inflammatory disorders among SCAD patients (< 5% in most cohorts), but this may be underdiagnosed [7]. Nevertheless, we believe there might be a potential association between autoimmune diseases and SCAD, particularly in younger women who are more susceptible to autoimmune diseases. Given the rarity of both the autoimmune disease APS and SCAD, it is important to consider the diagnostic aspect, as there may be a plausible link between the two [8, 9].

Several non-thrombotic manifestations, not included in the diagnostic criteria for APS, have been described in recent years. These include fibromuscular dysplasia, thrombocytopenia, valve disease, microthrombotic nephropathy, vasculitis and cognitive dysfunction. Limited data are available on how to treat these non-thrombotic manifestations and whether they should be incorporated into the diagnostic criteria [1]. We propose that SCAD could be another of these rarer manifestations.

Due to SCAD´s association with medial dissection rather than atherosclerotic plaque, performing PCI is challenging and is associated with poorer short- and long-term outcomes compared with PCI involving atherosclerotic lesions. Medically managed SCAD lesions often exhibit angiographic improvement, characterized by restored blood flow and reduced stenosis severity. Based on this, over 80% of patients can achieve success with conservative treatment [7] and expert consensus recommends a drug-based approach over immediate revascularization in clinically stable patients (algorithm). While SCAD may manifest in other coronary arteries beyond the initial dissection, revascularization has not been proven to prevent recurrent SCAD and myocardial infarction [7].

Selecting the appropriate treatment for a patient experiencing acute myocardial infarction due to SCAD can be challenging. Various factors come into play, including the patient's clinical and hemodynamic status, SCAD localization, extent of myocardial damage, and anatomical conditions, as well as comorbidity [7]. This complexity is heightened in APS patients requiring anticoagulation with warfarin, further complicating treatment considerations.

Coronary artery bypass surgery (CABG) has been reported as a technically successful revascularization option for patients with SCAD. However, this procedure is limited to cases involving high-risk anatomical lesions, failed PCI attempts, or situations where PCI is deemed to be of high risk or insufficient for treating ongoing ischemia. Surgical revascularization is only recommended for a very small percentage (< 1%) of patients with acute SCAD. While the short-term success rate of CABG is high, the long-term success rate is poor due to recanalization of the original coronary arteries, leading to competing flow and subsequent graft occlusion [7, 10].

While revascularization may enhance blood flow in the dissected vessel, it does not protect against the risk of dissection extension, including to central areas of the coronary arteries. PCI may also lead to an increased incidence of hospital readmission after 30 days. Therefore, the current consensus recommends a hospitalization period of 3–5 days for patients with acute myocardial infarction due to SCAD to monitor for adverse ischemic events [7].

**Figure:**
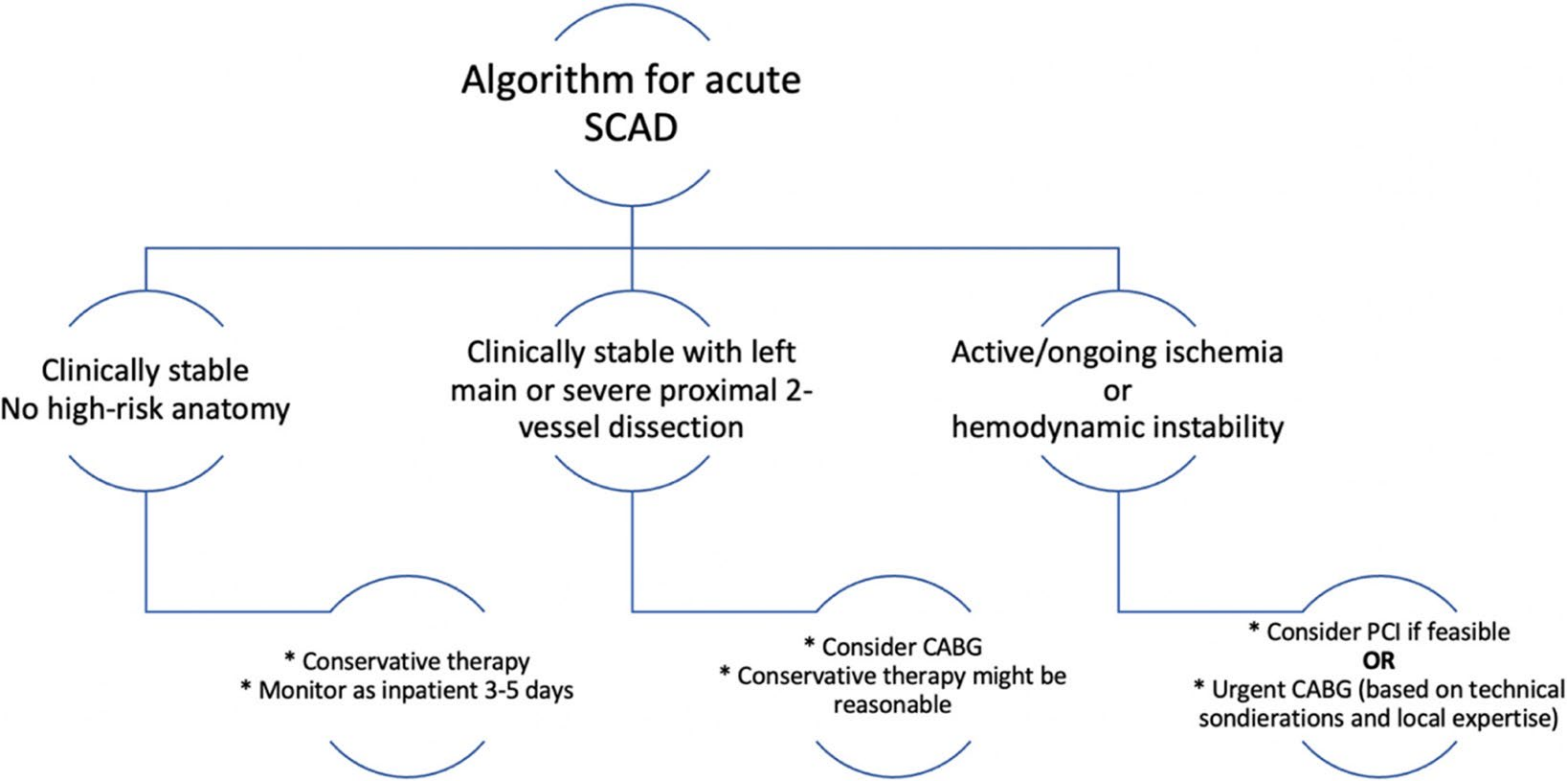
**Algorithm 1.** Treatment algorithm including revascularization in acute spontaneous coronary artery dissection (SCAD). Adapted from the American Heart Association [8]

Approximately 90% of patients diagnosed with SCAD are discharged with at least one antiplatelet agent. Although DAPT is recommended for patients undergoing PCI, there is a lack of clinical studies on the efficacy of platelet inhibitors in SCAD. Expert consensus suggests that DAPT treatment can be considered in the acute phase and continued for up to one year for patients receiving drug treatment. Treating APS patients with triple antithrombotic therapy, where anticoagulation is necessary, poses a challenge. In such cases, the duration of DAPT treatment should be kept as short as possible, and maintaining close INR control within the target range of 2–2.5 is crucial if a platelet inhibitor is added.

The evidence of statins is limited in SCAD as there is no atherosclerotic cause. However, according to guidelines, statins should be added in the treatment of hyperlipidemia in patients with coronary artery disease [7]. Notably, statins have been shown to reduce the occurrence of thrombi, particularly in patients with secondary APS [11].

Since our patient developed infarction sequelae and heart failure, he was initiated on ACE inhibitors and beta blocker following guidelines. Beta blockers may offer an additional benefit in preventing the recurrence of SCAD, showing up to a 64% reduction in the incidence of recurrent SCAD over a median of 3.1 years [4].

Our case illustrates the diagnostic challenges and atypical presentations associated with SCAD, particularly when complicated by APS. When the patient was initially admitted due to randomly measured elevated troponin and an INR above the therapeutic range, acute coronary syndrome was not immediately suspected. However, SCAD should be considered as a differential diagnosis, especially in younger patients with APS and elevated troponins, once other differential diagnoses have been ruled out. Hence, the possibility of vasculitis is low.

Microthrombosis is a common feature of APS, and referring APS patients with chest pain for cardiac evaluation and coronary angiography may be warranted. Although a definitive connection between SCAD and APS has not been established, further research in this area is needed to clarify potential links.

## Data Availability

Data of this patient´s journal is not accessible for the public.
